# Bis(2-amino­pyridine)dibenzoatocobalt(II). Erratum

**DOI:** 10.1107/S2056989016015504

**Published:** 2016-10-28

**Authors:** Wen-Zheng Ju, Rui-Hua Jiao, Ping Cao, Rui-Qin Fang

**Affiliations:** a Institute of Functional Biomolecules, State Key Laboratory of Pharmaceutical Biotechnology, Nanjing University, Nanjing 210093, People’s Republic of China

## Abstract

In the paper by Ju *et al.* [*Acta Cryst.* (2006), E**62**, m1012–m1013], the metal atom was reported incorrectly.

## Abstract 2

Erratum to *Acta Cryst.* (2006), E**62**, m1012—m1013.

The authors of Ju *et al.* (2007) ▸ have indicated that the metal atom in the reported complex was assigned incorrectly. The paper is therefore withdrawn from the published literature.
